# The TaSOC1‐TaVRN1 module integrates photoperiod and vernalization signals to regulate wheat flowering

**DOI:** 10.1111/pbi.14211

**Published:** 2023-11-08

**Authors:** Xumei Luo, Bingyan Liu, Li Xie, Ke Wang, Dengan Xu, Xiuling Tian, Lina Xie, Lingli Li, Xingguo Ye, Zhonghu He, Xianchun Xia, Liuling Yan, Shuanghe Cao

**Affiliations:** ^1^ Institute of Crop Sciences Chinese Academy of Agricultural Sciences (CAAS) Beijing China; ^2^ Department of Plant and Soil Sciences Oklahoma State University Stillwater OK USA

**Keywords:** *Triticum aestivum*, TaSOC1, TaVRN1, flowering time, photoperiod, vernalization

## Abstract

Wheat needs different durations of vernalization, which accelerates flowering by exposure to cold temperature, to ensure reproductive development at the optimum time, as that is critical for adaptability and high yield. TaVRN1 is the central flowering regulator in the vernalization pathway and encodes a MADS‐box transcription factor (TF) that usually works by forming hetero‐ or homo‐dimers. We previously identified that TaVRN1 bound to an MADS‐box TF TaSOC1 whose orthologues are flowering activators in other plants. The specific function of TaSOC1 and the biological implication of its interaction with TaVRN1 remained unknown. Here, we demonstrated that TaSOC1 was a flowering repressor in the vernalization and photoperiod pathways by overexpression and knockout assays. We confirmed the physical interaction between TaSOC1 and TaVRN1 in wheat protoplasts and *in planta*, and further validated their genetic interplay. A *Flowering Promoting Factor 1‐*like gene *TaFPF1‐2B* was identified as a common downstream target of *TaSOC1* and *TaVRN1* through transcriptome and chromatin immunoprecipitation analyses. TaSOC1 competed with TaVRT2, another MADS‐box flowering regulator, to bind to TaVRN1; their coding genes synergistically control *TaFPF1‐2B* expression and flowering initiation in response to photoperiod and low temperature. We identified major haplotypes of *TaSOC1* and found that *TaSOC1‐Hap1* conferred earlier flowering than *TaSOC1‐Hap2* and had been subjected to positive selection in wheat breeding. We also revealed that wheat *SOC1* family members were important domestication loci and expanded by tandem and segmental duplication events. These findings offer new insights into the regulatory mechanism underlying flowering control along with useful genetic resources for wheat improvement.

## Introduction

Flowering at the optimum time is critical for crop reproduction and yield formation. Control of flowering time (usually represented by heading date in crops) is an important target for crop breeding to harness adaptation to local environments and increase yield potential. The flexible regulatory machinery underlying the decision to flower enhances adaptability of crops to a broad range of agronomic and climatic conditions. Wheat (*Triticum aestivum*) is the most widely grown crop and provides staple foodstuff for approximately one‐third of the world population (FAO, https://www.idrc.ca/en/article/facts‐Figures‐food‐and‐biodiversity). Identification and functional dissection of flowering genes in wheat are therefore essential to improve environmental adaptability and yield potential.

The requirement for prolonged exposure to low temperature, known as vernalization, prevents plants from flowering under freezing conditions, especially during long winter in temperate regions (Kim *et al*., 2009). Wheat cultivars grown in different environments need diverse vernalization characteristics to ensure flowering initiation and reproductive development at the optimum time, as that is critical for high yield and crop rotations (Milec *et al*., 2014). Wheat cultivars are classified as winter and spring types based on vernalization requirements. *TaVRN1* is the central regulator in the vernalization flowering pathway and acts upstream of *TaVRN2* and *TaVRN3*, two other important flowering genes (Yan *et al*., 2003, 2004b, 2006). TaVRN1 directly binds to the *TaVRN2* promoter to repress its transcription (Chen and Dubcovsky, 2012; Deng *et al*., 2015; Yan *et al*., 2004a). TaVRN1 also alleviates repression of *TaVRN2* on *TaVRN3*, allowing *TaVRN3* to promote flowering (Li *et al*., 2011). Moreover, HvVRN1 activates *HvVRN3* expression by direct binding to its promoter in barley (*Hordeum vulgare*), a close relative of wheat (Deng *et al*., 2015). A recent study showed that TaVRN1 could initiate positive self‐regulation by interacting with TaVRT2 (Xie *et al*., 2021). *TaVRN1* can directly sense vernalization through chromatin remodelling (Diallo *et al*., 2012; Oliver *et al*., 2009). In addition, vernalization can promote the maturity and processing of the *TaVRN1* pre‐mRNA by TaGRP O‐GlcNAcylation (Xiao *et al*., 2014; Xu and Chong, 2018). *TaVRN1* is also activated by a vernalization‐induced long non‐coding RNA VAS together with transcription factor TaRF2b (Xu *et al*., 2021).

*TaVRN1* encoding a MADS‐box transcription factor (TF) is highly homologous to *AP1*, a floral meristem identity gene in *Arabidopsis* (Danyluk *et al*., 2003; Mandel *et al*., 1992; Trevaskis *et al*., 2003; Yan *et al*., 2003). MADS‐box TFs usually work by forming hetero‐ or homo‐dimers (de Folter *et al*., 2005; Kaufmann *et al*., 2005). Hence, identification of MADS‐box proteins interacting with TaVRN1 is extremely helpful to expand its regulatory network in flowering pathways. We previously identified a SUPPRESSOR OF OVEREXPRESSION OF CONSTANS1 (SOC1)‐like MADS‐box TF, herein designated as TaSOC1, as a partner of TaVRN1 through a yeast two‐hybrid library screening (Cao and Yan, 2013). In addition, Li *et al*. (2013) validated the interaction using pull‐down assays. In model plants, *SOC1* integrates multiple flowering signals, such as photoperiod, temperature, hormone and plant age (Cao *et al*., 2021; Hyun *et al*., 2016). *HvSOC1‐like1* in barley also responds to vernalization (Papaefthimiou *et al*., 2012). These findings suggested that *SOC1*‐like genes of temperate cereals might be involved in vernalization‐induced flowering. However, the function of *SOC1* homologues remains largely unknown in wheat.

Considering that TaSOC1 can interact with TaVRN1, the central factor in vernalization flowering pathway, it is necessary to elucidate the specific function of *TaSOC1* and its genetic relationship with *TaVRN1* in flowering initiation. In the present study, *TaSOC1* was validated as a flowering repressor in the vernalization and photoperiod pathways. We confirmed the interaction of TaSOC1 and TaVRN1 both physically and genetically. We also identified *Flowering Promoting Factor 1*‐like gene *TaFPF1‐2B* as a common downstream target of *TaSOC1* and *TaVRN1*. Moreover, TaSOC1 could impair the interaction between TaVRN1 and TaVRT2, another MADS‐box flowering promoter, and hereby a model underpinning the synergistic flowering regulation in response to vernalization and photoperiod was proposed. We further identified natural variations at *TaSOC1* and its orthologues, and examined their genetic effects on flowering time in wheat cultivars. These results not only improve understanding of the regulatory network underlying flowering initiation but also provide important genetic resources for wheat breeding.

## Results

### *TaSOC1* is a flowering repressor in the vernalization pathway

**Figure 1:**
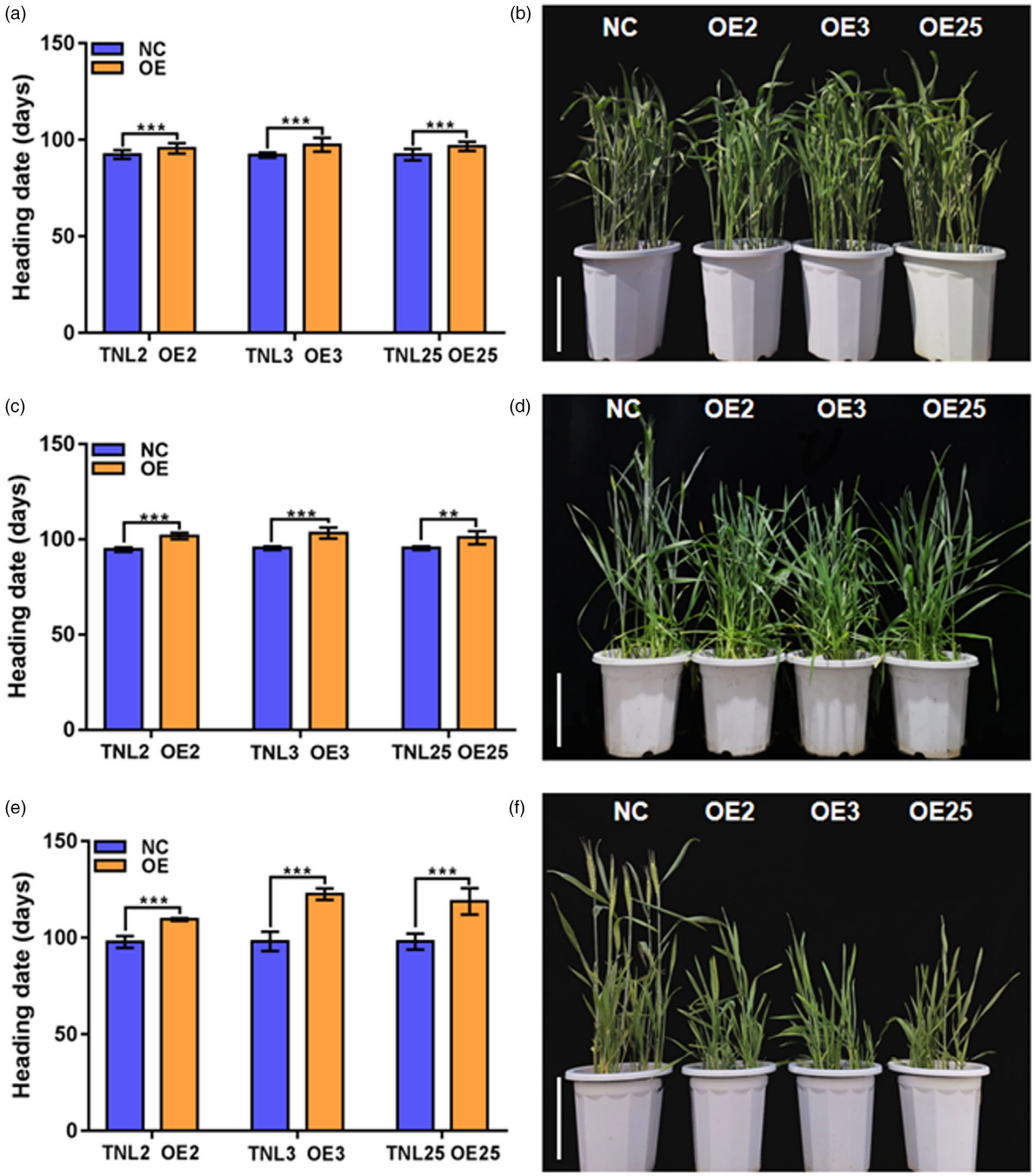
Phenotypic analyses of *TaSOC1* overexpression lines (*TaSOC1*‐OE) and transgenic null lines (TNL) under different vernalization conditions. Statistical analyses of the heading date of *TaSOC1*‐OE and TNL after exposure to 30‐ (a), 14‐ (c) and 0‐day (e) of vernalization treatments (*n* = 40 plants). OE2, OE3 and OE25 are representative lines of *TaSOC1*‐OE. TNL is the negative control (NC). ***P < 0.001. Phenotypic display of heading date of *TaSOC1*‐OE and TNL after exposure to 30‐ (b), 14‐ (d) and 0‐day (f) vernalization treatments. Scale bar, 30 cm.

To determine the function of *TaSOC1*, we generated overexpression (OE) lines in winter wheat cultivar Kenong 199 (KN199). Thirty‐three independent positive transgenic lines were created and three representative lines, *TaSOC1*‐OE2, *TaSOC1*‐OE3 and *TaSOC1*‐OE25 were used in subsequent analyses. Phenotypic analyses showed that *TaSOC1* overexpression significantly delayed flowering under non‐vernalization, incomplete vernalization (4 °C for 14 days) and complete vernalization (4 °C for 30 days) conditions (Figure 1a–f). Remarkably, *TaSOC1* inhibited flowering more significantly under non‐vernalization conditions than after vernalization treatments (Figure 1a–f). *TaSOC1* overexpression lines (*TaSOC1*‐OE) flowered approximately 4.3 days later than transgenic‐null lines (TNL) under complete vernalization conditions, whereas *TaSOC1* overexpression delayed flowering 6.9 and 20.8 days under incomplete vernalization and non‐vernalization conditions, respectively (Dataset S1).

**Figure 2:**
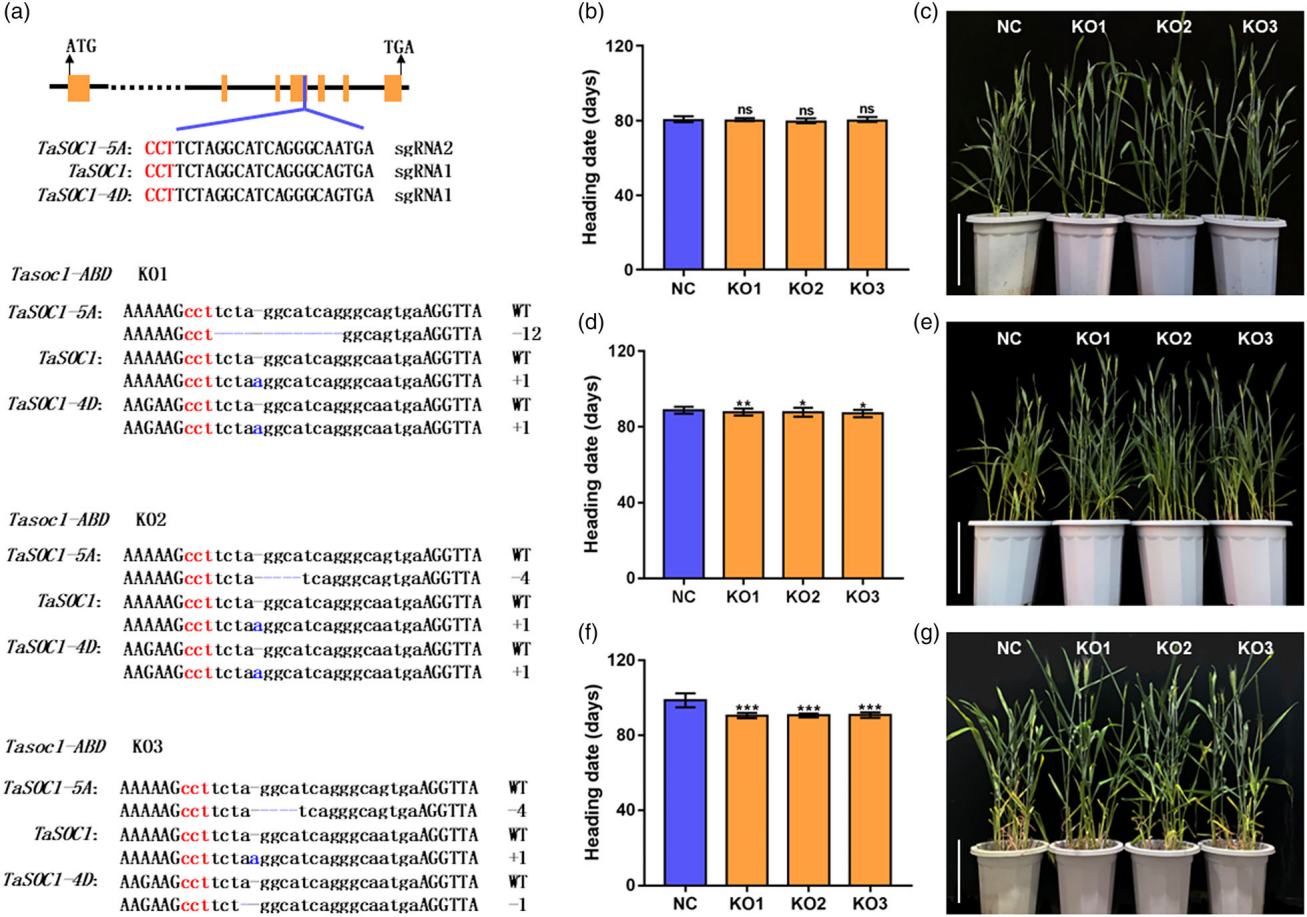
*TaSOC1* knockout lines (*TaSOC1*‐KO) and phenotypic investigation of *TaSOC1*‐KO and KN199 under differing vernalization conditions. (a) Sequencing‐based identification of *TaSOC1* knockout (KO) mutants. PAM and mutant sites are shown in red and blue, respectively; orange bars and black lines represent exons and introns, respectively; ATG, start codon; sgRNA, small guide RNA; TGA, stop codon. Statistical analyses (b) and phenotype display (c) of the heading date of *TaSOC1*‐KO and KN199 following 30 days (complete) of vernalization. Statistical analyses (d) and phenotype display (e) of heading dates of *TaSOC1*‐KO and KN199 following 14 days (partial) vernalization. Statistical analyses (f) and phenotype display (g) of heading dates of *TaSOC1*‐KO and KN199 under non‐vernalization conditions (*n* = 40 plants). KO1, KO2 and KO3 are representative *TaSOC1*‐KO. KN199 was used as the negative control (NC). *P < 0.05, **P < 0.01, ns, not significant; scale bar, 30 cm.

*TaSOC1*, a *SOC1*‐like gene on chromosome 4B, has orthologues, temporarily designated *TaSOC1‐5A* and *TaSOC1‐4D*, on chromosomes 5A and 4D. Considering that orthologous genes usually have functional redundancy in wheat, we created triple knockout lines of the *SOC1*‐like genes (*TaSOC1*‐KO) in KN199. Phenotypic investigation showed that *TaSOC1*‐KO lines had similar heading date with KN199 under complete vernalization conditions, whereas *TaSOC1*‐KO significantly accelerated flowering approximately 1.0 and 8.0 days under incomplete and non‐vernalization conditions, respectively (Figure 2a–g; Dataset S2). This result confirmed that *TaSOC1* acted as a flowering repressor in the vernalization pathway.

### TaSOC1 physically interacts with TaVRN1

**Figure 3:**
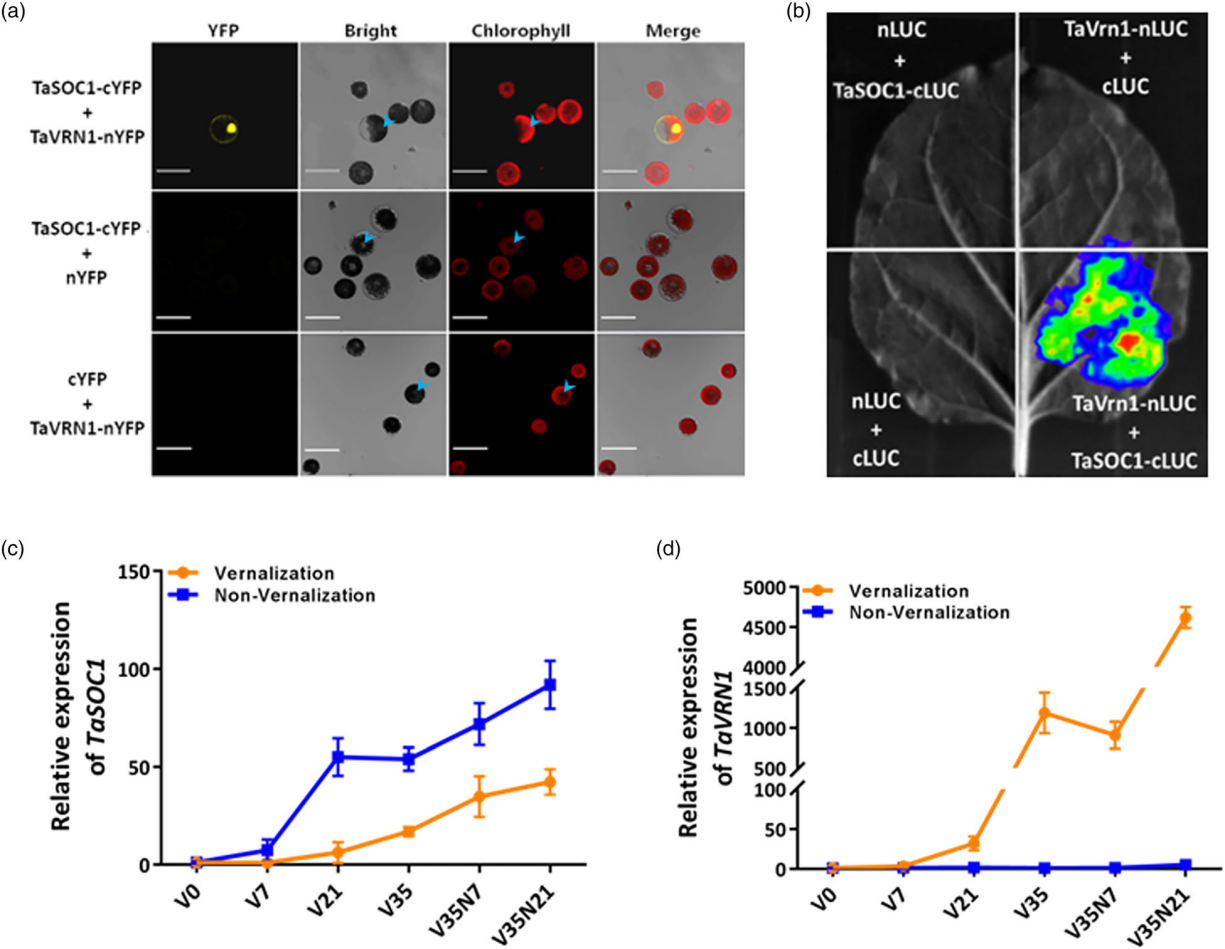
Confirmation of the interaction between TaSOC1 and TaVRN1, and spatiotemporal overlapping of their expression responses to vernalization in leaves. Bimolecular fluorescence complementation (BiFC) (a) and luciferase complementation imaging (LCI) (b) assays confirm interaction of TaSOC1 and TaVRN1 in wheat protoplasts and *N. benthamiana* leaves, respectively. TaSOC1‐cYFP+nYFP and cYFP+TaVRN1‐nYFP were used as negative controls in BiFC assays; nLUC+TaSOC1‐cLUC, TaVRN1‐nLUC+cLUC and nLUC+cLUC were used as negative controls in LCI assays; the nuclei of some cells in the bright and chlorophyll fields of BiFC images were labelled using arrows. Scale bars, 50 μm; LUC, luciferase. *TaSOC1* (c) and *TaVRN1* (d) had clear responses to vernalization treatments (*n* = three biological replicates). Sampling time‐points include the day before vernalization (V0), 7th day (V7), 21st day (V21) and 35th day of vernalization (V35), and 7th day (V35N7) and 21st day post‐vernalization (V35N21); orange and blue lines represent vernalization and non‐vernalization (negative control) treatments, respectively; error bars indicate standard deviations of three biological replicates.

The MADS‐box TF TaSOC1 was identified as a partner of TaVRN1 by Y2H assays in a previous study (Cao and Yan, 2013). To confirm the interaction, we firstly performed a bimolecular fluorescence complementation (BiFC) assay and found that TaVRN1 and TaSOC1 could bind with each other in wheat protoplasts (Figure 3a). Their interaction signals appeared mainly in the nuclei, suggesting that TaVRN1 and TaSOC1 formed a transcriptional complex to regulate downstream genes. We also employed luciferase complementation imaging (LCI) assays to test the interaction between TaSOC1 and TaVRN1 in leaves of *Nicotiana benthamiana*. Significant luciferase (LUC) signals were detected in the area where TaSOC1‐cLUC and TaVRN1‐nLUC were co‐infiltrated, whereas there were no LUC signals in the negative controls (Figure 3b), confirming the interaction of TaVRN1 and TaSOC1 *in planta*.

We investigated the expression patterns of *TaVRN1* and *TaSOC1* in Wheat Expression Browser (http://wheat‐expression.com/), which showed that both genes were highly expressed in leaves, the most important tissue to response to environmental cues for flowering initiation (Figure S1a,b). Considering that TaVRN1 is the central regulator in the vernalization flowering pathway and interacts with TaSOC1, we compared their response to vernalization. Reverse transcription quantitative PCR (RT‐qPCR) assays showed that *TaVRN1* and *TaSOC1* had high expression activity in leaves of KN199 and also significantly responded to vernalization (Figure 3c,d). Strikingly, vernalization induced the expression of *TaVRN1* but repressed expression of *TaSOC1* (Figure 3c,d). These results indicated that *TaVRN1* and *TaSOC1* had an overlapping spatiotemporal expression window to initiate flowering in the vernalization pathway.

### *TaSOC1* probably has a genetic interaction with *TaVRN1* in the vernalization flowering pathway

**Figure 4:**
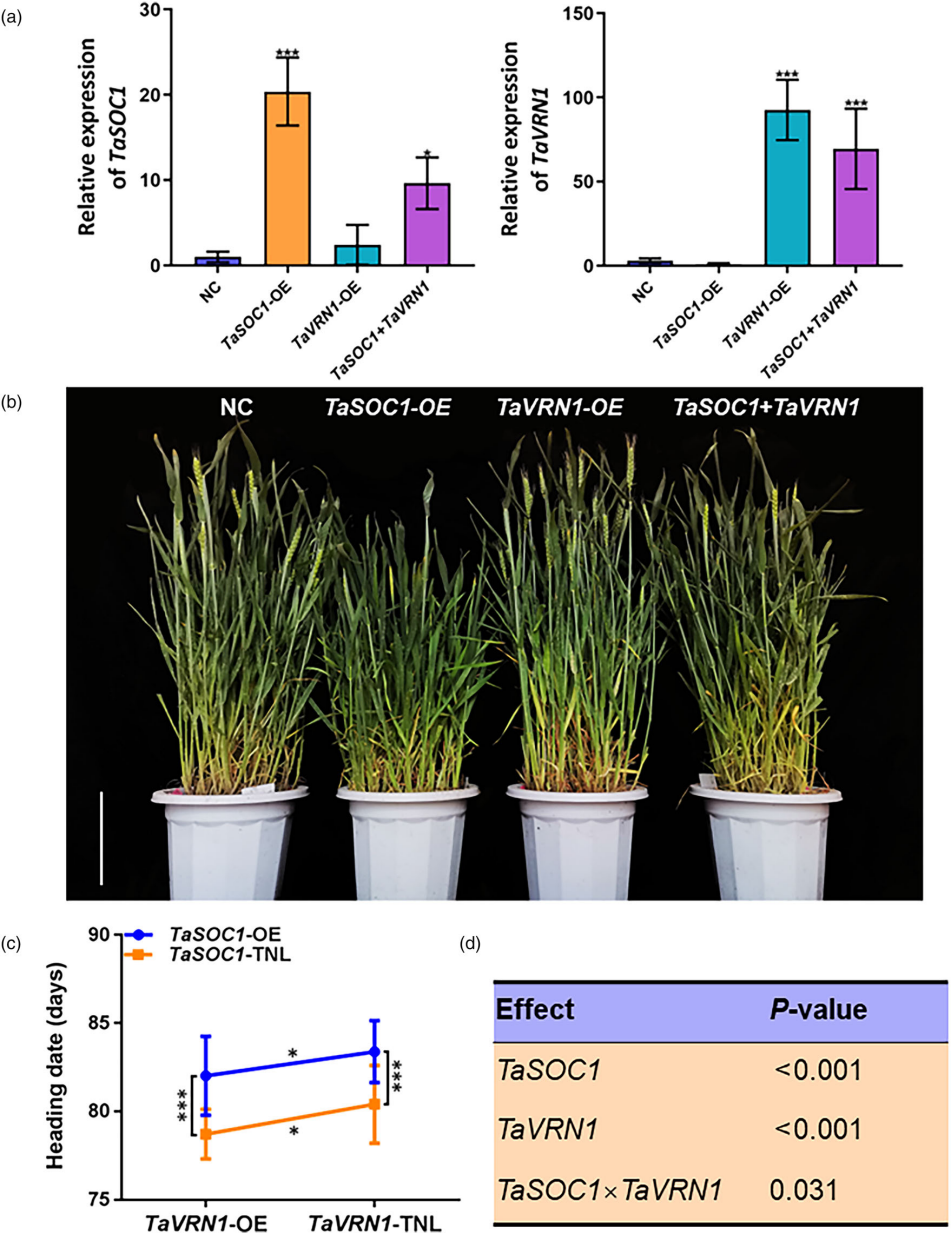
Identification of genetic interaction between *TaSOC1* and *TaVRN1*. (a) Relative expression levels of *TaSOC1* and *TaVRN1* in the lines with different genotypes in a segregating population derived from cross *TaSOC1*‐OE × *TaVRN1*‐OE. Transgenic null lines (TNL) are negative control (NC); *TaSOC1*‐OE, *TaSOC1* overexpression lines; *TaVRN1*‐OE, *TaVRN1* overexpression lines; *TaSOC1* + *TaVRN1*, double overexpression lines. (b) Phenotype visualization for heading dates of different genotypes under incomplete vernalization conditions (14‐day vernalization treatments). Scale bar, 30 cm. Student's *t* tests (c) and two‐way ANOVA (d) for heading dates for different genotypes under incomplete vernalization conditions (*n* = 40 plants). *P < 0.05, ***P < 0.001; bars represent standard deviations of three biological replicates.

To further validate the interaction of *TaSOC1* and *TaVRN1* in the vernalization flowering pathway, we created a segregating population from a cross of *TaSOC1*‐OE and *TaVRN1* overexpression lines (*TaVRN1*‐OE). We genotyped individuals in the population to identify *TaSOC1*‐OE, *TaVRN1*‐OE, their double OE lines and NC (negative control). RT‐qPCR assays showed that overexpressed *TaSOC1* and *TaVRN1* in *TaSOC1* + *TaVRN1* lines had similar expression levels to their counterparts in *TaSOC1*‐OE and *TaVRN1*‐OE lines (Figure 4a). Compared to non‐vernalization, incomplete vernalization promoted flowering of *TaSOC1*‐OE, *TaVRN1*‐OE, *TaSOC1* + *TaVRN1* and NC (Dataset S1). Moreover, the plants in each line had more consistent flowering time under incomplete vernalization conditions than under non‐vernalization conditions, so we investigated flowering time for a genetic analysis under incomplete vernalization conditions. Phenotypic investigation revealed that the *TaSOC1* + *TaVRN1* flowered earlier than *TaSOC1*‐OE and later than *TaVRN1*‐OE (Figure 4b; Dataset S3). Statistical analyses showed that both *TaSOC1* and *TaVRN1* had significant effects on flowering time (*P* < 0.05) in the segregating population (Figure 4c,d). A significant genetic epistatic effect (*P* < 0.05) between *TaSOC1* and *TaVRN1* was also detected (Figure 4d).

### *Flowering Promoting Factor 1*‐like gene *TaFPF1‐2B* is a common downstream target of *TaSOC1* and *TaVRN1*

**Figure 5:**
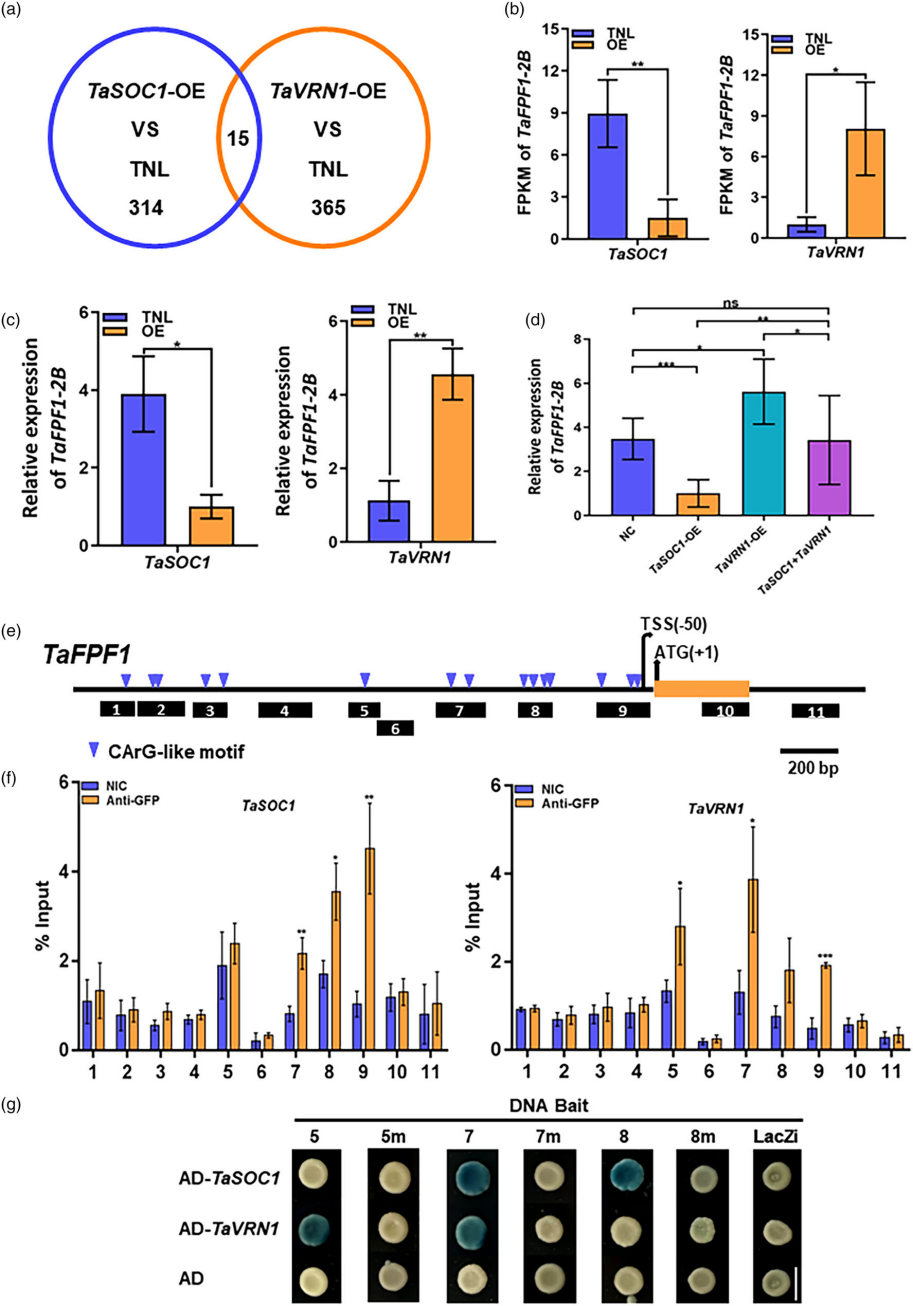
Identification of common downstream target genes regulated by *TaSOC1* and *TaVRN1* and detection of TaSOC1 and TaVRN1 enrichment at different sites of *TaFPF1*. (a) Identification of differentially expressed genes (DEGs) in *TaSOC1* overexpression lines (*TaSOC1*‐OE) and *TaVRN1* overexpression lines (*TaVRN1*‐OE) compared to respective transgenic null lines (TNL) based on RNA‐seq assays. The overlapping part of the blue and orange circles indicates 15 DEGs shared by *TaSOC1* and *TaVRN1*. RNA‐seq (b) and reverse transcription quantitative PCR (RT‐qPCR) (c) assays for expression levels of *TaFPF1‐2B* in *TaSOC1*‐OE and *TaVRN1*‐OE (*n* = three biological replicates). FPKM, fragments per kilobase million. OE, overexpression line; TNL, transgenic null line. Note: *TaFPF1* expression levels in *TaSOC1*‐OE and *TaVRN1*‐OE were compared to the counterparts of their respective TNLs. (d) *TaFPF1‐2B* expression analyses in a F_2_ segregating population derived from cross *TaSOC1*‐OE × *TaVRN1*‐OE using RT‐qPCR (*n* = three biological replicates). NC, negative control (TNLs identified from the F_2_ population). (e) Schematic of detection sites in the *TaFPF1* promoter. The blue arrowheads indicate the positions of CArG‐like motifs, and black boxes 1–11 represent different detection sites; orange box, first exon; TSS, transcription start site; ATG, start codon. (f) TaSOC1 and TaVRN1 enrichment at different sites of the *TaFPF1* promoter using chromatin immunoprecipitation quantitative PCR (ChIP‐qPCR) assays (*n* = three biological replicates). Anti‐GFP, GFP antibody; NIC, non‐immune control; *P < 0.05, **P < 0.01, ***P < 0.001, ns, not significant. (g) Validation of TaSOC1 and TaVRN1 binding to the representative sites 5, 7 and 8, close to transcription sites of *TaFPF1*, by yeast one‐hybrid (Y1H) assays. 5, 7 and 8 indicate the sites 5, 7 and 8 in the *TaFPF1* promoter; 5 m, 7 m and 8 m represent their respective mutated versions. The promoter in LacZi empty vector is used as a negative DNA bait. AD represents activation domain in the vector pB42AD.

Since TaSOC1 interacts with TaVRN1 physically and genetically, they can form a complex to regulate common downstream flowering genes. We conducted transcriptome analyses to expand the regulatory network underlying the cooperative modulating flowering controlled by *TaVRN1* and *TaSOC1*. Both TaVRN1 and TaSOC1 caused differential expression of many genes in leaves at the beginning of stem elongation (floret differentiation), a key spatiotemporal window of flowering initiation in response to environmental cues (Xie *et al*., 2021). In total, 365 and 314 (|Log_2_(Fold change)| >1 and *P* < 0.01) differentially expressed genes (DEGs) were detected in the *TaVRN1*‐OE and *TaSOC1*‐OE, compared with their respective TNL (Figure 5a; Datasets S4 and S5). Fifteen of those genes were common downstream genes of *TaVRN1* and *TaSOC1*, indicating some proportion of overlap in their downstream targets at the initial stage of flowering (Dataset S6). Interestingly, a *Flowering Promoting Factor 1*‐like gene *TaFPF1‐2B* (*TraesCS2B02G059500*) was identified as a common downstream target of *TaVRN1* and *TaSOC1*. *Flowering Promoting Factor 1*‐like genes were validated as flowering accelerators in the photoperiod and GA pathways in *Arabidopsis* (Kania *et al*., 1997; Melzer *et al*., 1999), and overexpression of *NtFPF1* promotes flowering in *Nicotiana* plants (Smykal *et al*., 2004). *TaFPF1‐2B* was significantly up‐regulated in *TaVRN1*‐OE and downregulated in *TaSOC1*‐OE, which was confirmed by RT‐qPCR assays (Figure 5b,c). We also investigated the expression of *TaFPF1‐2B* in the segregating population derived from the cross between *TaSOC1*‐OE and *TaVRN1*‐OE. *TaSOC1* and *TaVRN1* showed significant interaction effects on expression of *TaFPF1‐2B* (Figure 5d). Thus, TaSOC1 and TaVRN1 probably orchestrate expression of *TaFPF1‐2B* to modulate flowering.

### TaSOC1 and TaVRN1 have overlapping binding sites in the *TaFPF1‐2B* promoter

We conducted chromatin immunoprecipitation quantitative PCR (ChIP‐qPCR) assays to determine whether *TaFPF1‐2B* is a direct downstream target of *TaSOC1* and *TaVRN1*. MADS‐box transcription factors such as TaVRN1 and TaSOC1, were shown to bind with CArG motifs within the promoters of their target genes (Dubcovsky *et al*., 2008; Kane *et al*., 2007; Theißen and Gramzow, 2016; Xie *et al*., 2021). We thus designed primer pairs to query the enrichment of TaSOC1 and TaVRN1 over 11 regions of the *TaFPF1‐2B* promoter covering the CArG‐like motifs in the respective overexpression lines carrying *TaSOC1*‐ or *TaVRN1*‐*YFP* fusion constructs (Figures 5e and S2). TaSOC1 and TaVRN1 were both significantly enriched at three sites containing CArG‐like motifs near the transcription start site (TSS) in the *TaFPF1‐2B* promoter (Figure 5f). Moreover, TaSOC1 and TaVRN1 shared two binding sites, suggesting that they could coordinately modulate *TaFPF1‐2B* expression through direct binding with its promoter.

We further used yeast one‐hybrid (Y1H) to validate the interaction of TaSOC1 and TaVRN1 with the representative sites 5, 7, 8 and 9 that are close to transcription start site of *TaFPF1* (Figures 5e and S2), for determining target sites and possible competition. The site 9 can generate auto‐activation in yeast, so we cannot identify its binding to TaSOC1 and TaVRN1 in Y1H. Notably, TaSOC1 and TaVRN1 specifically bind to the sites 8 and 5, respectively, and both can interact with the site 7 (Figure 5g). The mutated sites cannot bind to TaSOC1 and TaVRN1 (Figure 5g). The results of Y1H are consistent with those of ChIP‐qPCR assays and show that TaSOC1 and TaVRN1 may competitively bind to the site 7.

### *TaSOC1* is a flowering repressor in the photoperiod pathway

**Figure 6:**
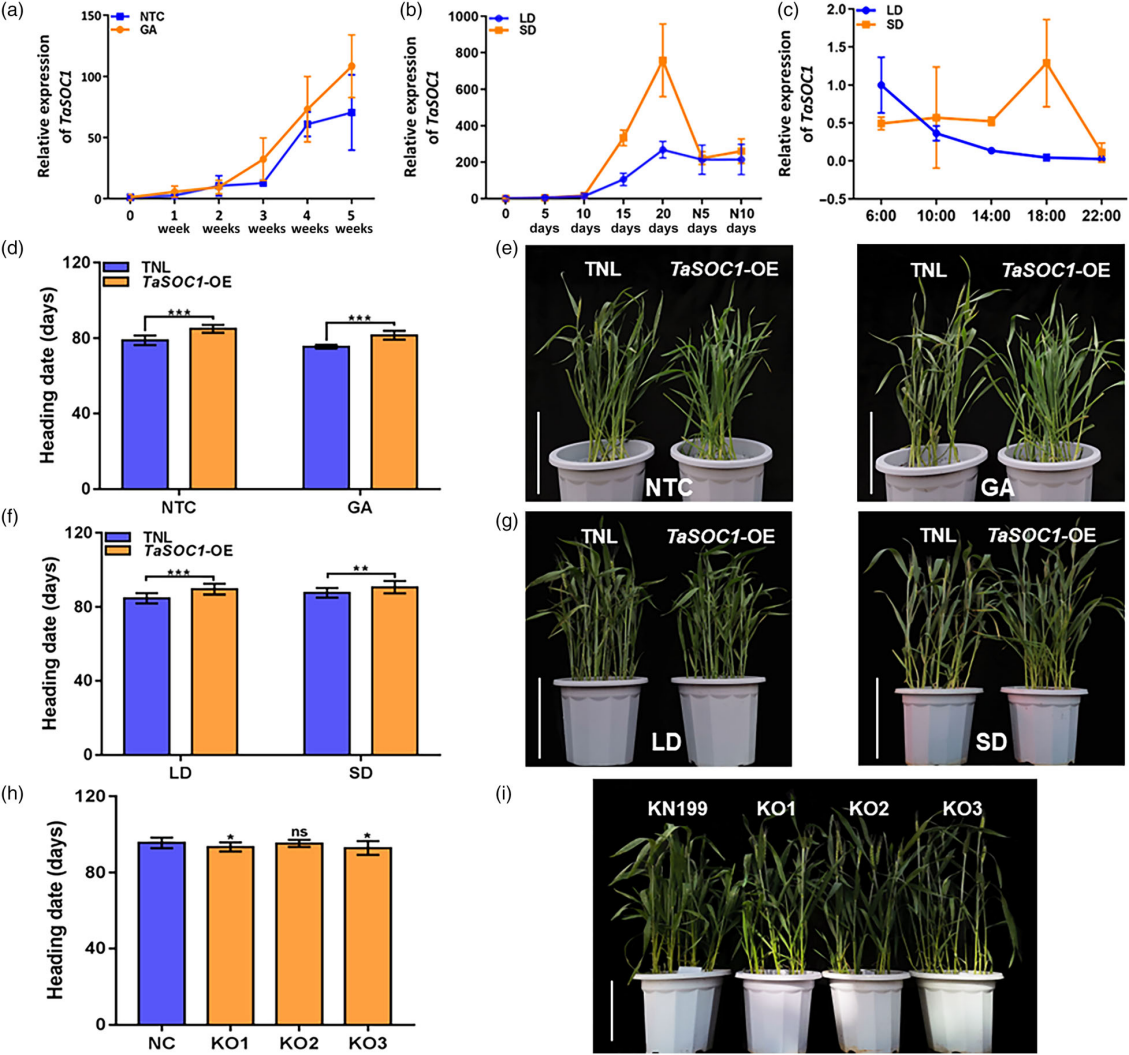
*TaSOC1* responses to gibberellin and photoperiod using quantitative real‐time PCR and transgenic assays. (a) Expression pattern of *TaSOC1* under GA treatments. Sampling time‐points include the day before GA treatment (0), and first (1 week), second (2 weeks), third (3 weeks), fourth (4 weeks) and fifth (5 weeks) week of GA treatment (*n* = three biological replicates); the orange and blue lines represent the GA treatments and non‐treatment controls (NTC), respectively. (b) Expression pattern of *TaSOC1* under photoperiod treatments (*n* = three biological replicates). The numbers on the horizontal axis represent sampling time‐points (0 shows the day before photoperiod treatments; 5, 10, 15 and 20 days represent the 5th, 10th, 15th and 20th day of photoperiod treatment, respectively; N5 days and N10 days indicate the 5th and 10th day after 20‐day photoperiod treatment, respectively); orange and blue lines represent short‐day (SD) and long‐day (LD) treatments, respectively. (c) Expression pattern of *TaSOC1* at different time‐points over a single day during photoperiod treatments (*n* = three biological replicates). Sampling time‐points include 6:00, 10:00, 14:00, 18:00 and 22:00 o'clock in 1 day; orange and blue lines represent SD and LD treatments, respectively. Statistical analysis (d) and phenotypes (e) of heading date of *TaSOC1* overexpression lines (*TaSOC1*‐OE) and transgenic null lines (TNL) following GA treatments (*n* = 40 plants). Statistical analyses (f) and phenotypes (g) of heading date of *TaSOC1*‐OE and TNL under photoperiod treatments (*n* = 40 plants). Statistical analysis (h) and phenotypes (i) of heading date of *TaSOC1* knockout lines (*TaSOC1*‐KO) and KN199 under SD conditions following complete vernalization (*n* = 40 plants). KO1, KO2 and KO3, representative *TaSOC1*‐KO lines; NC (negative control), KN199; *P < 0.05, **P < 0.01, ***P < 0.001, ns, not significant; scale bar, 30 cm.

In addition to response to vernalization, *SOC1* can integrate photoperiod and gibberellin (GA) signals to regulate flowering in model plants (Lee *et al*., 2004; Moon *et al*., 2003; Ryu *et al*., 2009). To further uncover the function of *TaSOC1* in flowering initiation, we first investigated its dynamic expression pattern in leaves of KN199 under GA treatment. The foliage spray of GA significantly promoted seedling growth of KN199, demonstrating the effectiveness of GA treatments. However, there was no significant response in *TaSOC1* expression to GA (Figure 6a). We then compared the expression profiles of *TaSOC1* under different photoperiod treatments and found that *TaSOC1* in completely vernalized KN199 had higher expression levels under short‐day (SD) conditions than long‐day (LD) conditions (Figure 6b). The time‐course transcriptional variation of *TaSOC1* over a full day under different photoperiod conditions also showed that SD up‐regulated *TaSOC1* expression (Figure 6c). Phenotypic investigation also showed that GA treatments have similar effects on flowering time of *TaSOC1*‐OE and TNL (Figure 6d,e; Dataset S7). By contrast, photoperiod treatment had little effect on *TaVRN1* expression (Figure S3a,b). Upon being completely vernalized, *TaVRN1*‐OE had similar flowering time to TNL under LD or SD conditions (Figure S3c,d), whereas *TaSOC1*‐OE had significantly delayed flowering time compared to TNL regardless of day length (Figure 6f,g). Remarkably, *TaSOC1*‐OE flowered 4.9 days later than TNL under LD conditions, but were only 3.0 days under SD conditions, suggesting that up‐regulation of native *TaSOC1* in SD could buffer the effects of its transgenic overexpression on flowering time in a dose‐dependent way (Dataset S7). We further investigated the flowering time of *TaSOC1*‐KO and KN199 under SD conditions and found that two of the three *TaSOC1*‐KO lines had significantly earlier flowering time than KN199 (Figure 6h,i; Dataset S8). No significant difference in flowering time was observed between *TaSOC1*‐KO and KN199 under LD conditions (Figure 2b,c). Collectively, *TaSOC1* plays a key role in repressing wheat flowering under SD conditions.

### Natural variation at *TaSOC1* affects flowering time

**Figure 7:**
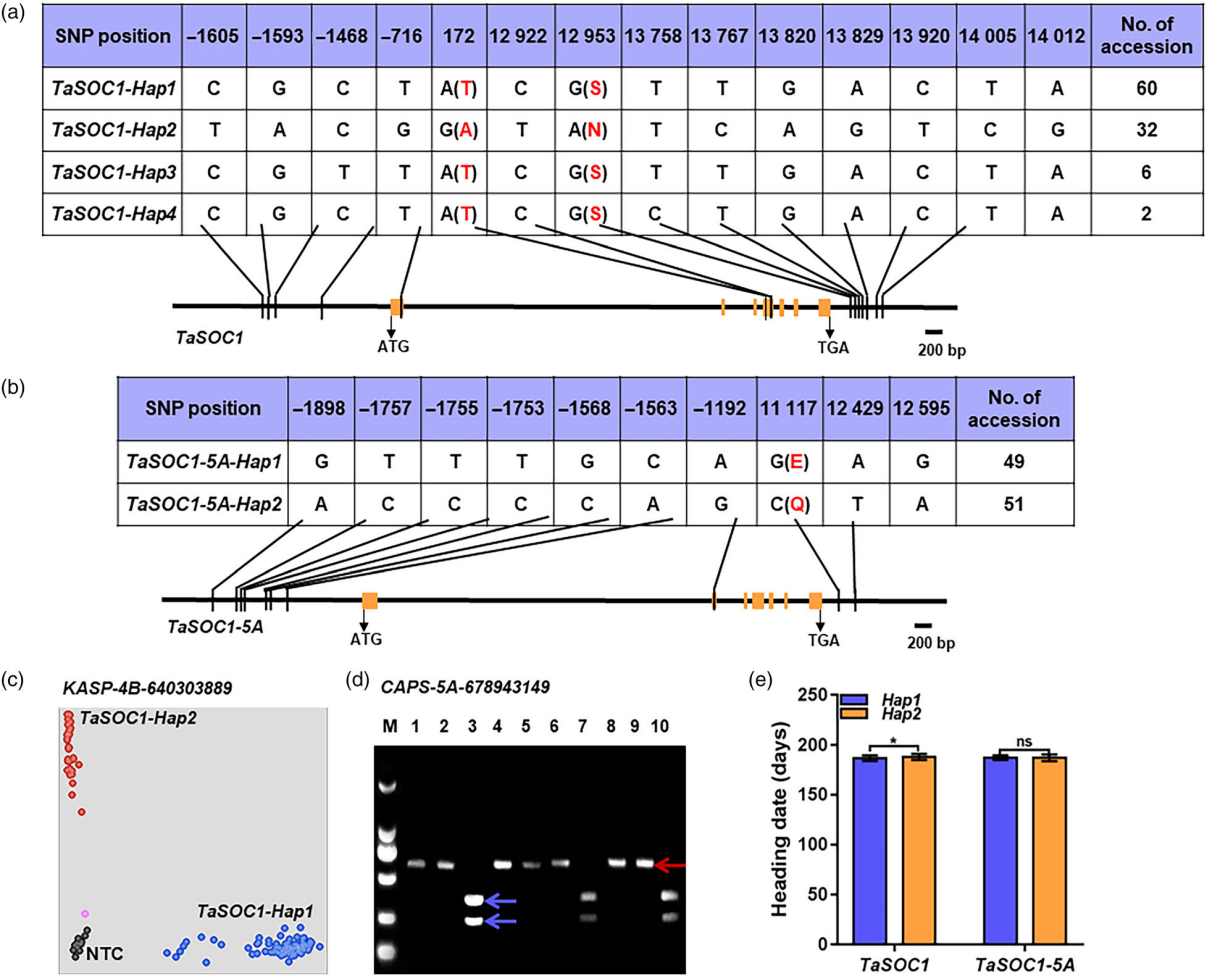
Allelic variations and genetic effects of *TaSOC1* and orthologue *TaSOC1‐5A*. Identification of sequence variants and haplotypes of *TaSOC1* (a) and *TaSOC1‐5A* (b). The amino acid changes are indicated in red; orange boxes show exons and black lines indicate promoters or introns; the first nucleotide of the start codon is defined as +1; ATG, start codon; TGA, stop codon. Visualization of genotyped gene‐specific markers for major haplotypes of *TaSOC1* (c) and *TaSOC1‐5A* (d). Blue, red and black dots in (c) indicate *TaSOC1‐Hap1*, *TaSOC1‐Hap2* and NTC (non‐template control) genotypes, respectively; the pink dot represents a cultivar that failed successful genotyping. In (d): M, DL2000 DNA marker ladder (TaKaRa); lanes 3, 7 and 10 indicate *TaSOC1‐5A‐Hap1* with the 246 and 372 bp target bands (blue arrows); lanes 1, 2, 4, 5, 6, 8 and 9 show the 618 bp target band (red arrow) for *TaSOC1‐5A‐Hap2*; bp, base pairs. (e) Genetic effects of the major *TaSOC1* and *TaSOC1‐5A* haplotypes on heading date. *P < 0.05; ns, not significant.

Since *TaSOC1* is a flowering regulator, it is necessary to investigate its genetic effect on flowering time in wheat breeding. We first identified sequence variants at *TaSOC1* and its orthologues *TaSOC1‐5A* and *TaSOC1‐4D* using the whole‐genome resequencing databases in WheatUnion (http://wheat.cau.edu.cn/WheatUnion/). *TaSOC1* and *TaSOC1‐5A* harboured polymorphic sites in their open reading frames, and 2 kb promoters as well as 1 kb 3′ untranslated regions (UTRs), whereas no sequence variation was detected in the corresponding regions of *TaSOC1‐4D*. Further analyses revealed that each of *TaSOC1* and *TaSOC1‐5A* had two major haplotypes, designated as *TaSOC1‐Hap1*, *TaSOC1‐Hap2*, *TaSOC1‐5A‐Hap1* and *TaSOC1‐5A‐Hap2*, based on their polymorphisms (Figure 7a,b; Dataset S9). A Kompetitive Allele‐Specific PCR (KASP) marker for the major haplotypes of *TaSOC1* was developed to genotype a natural population including 166 elite wheat cultivars from the Yellow and Huai River Valleys, the largest wheat zone in China (Figure 7c; Dataset S10). Association analyses showed that *TaSOC1‐Hap1* conferred significantly earlier flowering time than *TaSOC1‐Hap2* (Figure 7e; Dataset S11). A cleaved amplified polymorphic sequence (CAPS) marker for the haplotypes of *TaSOC1‐5A* was developed and used to genotype the natural population (Figure 7d; Dataset S10). However, no significant difference on flowering time was detected between *TaSOC1‐5A‐Hap1* and *TaSOC1‐5A‐Hap2* (Figure 7e, Dataset S11). The frequency (43.5%) of *TaSOC1‐5A‐Hap1* was comparable to that (56.5%) of *TaSOC1‐5A‐Hap2*. By contrast, *TaSOC1‐Hap1* (72.7%) had much higher distribution frequency than *TaSOC1‐Hap2* (27.3%), suggesting that the former had been subjected to positive selection in wheat breeding.

## Discussion

### Functional conservation and variation of *SOC1* homologues in modulating flowering

The majority of studied flowering plants have *SOC1*‐like genes (Cao *et al*., 2021). *SOC1* is a key flowering promoter in *Arabidopsis* (Hyun *et al*., 2016; Onouchi *et al*., 2000; Samach *et al*., 2000) and many *SOC1* homologues have also been identified as flowering activators in other plants, such as rice (*Oryza sativa*) (Lee *et al*., 2004), soybean (*Glycine max*) (Na *et al*., 2013; Zhong *et al*., 2012), maize (*Zea mays*) (Zhao *et al*., 2014), bamboo (*Phyllostachys heterocycla*) (Hou *et al*., 2021), mustard (*Sinapis alba*) (Borner *et al*., 2000), *Chrysanthemum* (Fu *et al*., 2013), *Eriobotrya japonica* (Jiang *et al*., 2019), *Medicago truncatula* (Fudge *et al*., 2018), *Petunia* (Ferrario *et al*., 2004) and *Phyllostachys violascens* (Liu *et al*., 2016). Rice *OsMADS50*/*OsSOC1* and *OsMADS56* have the highest similarity with *SOC1* in *Arabidopsis*. *OsMADS50* is up‐regulated during the floral transition and its overexpression leads to extremely early flowering (Lee *et al*., 2004). *OsMADS50* is expressed in vegetative tissues with elevated expression at the time of floral initiation and its overexpression in *Arabidopsis* also accelerates flowering (Lee *et al*., 2004; Tadege *et al*., 2003). However, *OsMADS56* functions as a flowering repressor (Ryu *et al*., 2009). Here, we showed that *TaSOC1* behaved as a flowering repressor. Phylogenetic analyses across major cereal crops and *Arabidopsis* showed that *TaSOC1* was homologous to *SOC1*, *OsMADS50* and *OsMADS56* (Figure S4a). Synteny analysis revealed that *TaSOC1* was orthologous to *OsMADS50* (Figure S4b). Since *OsMADS50* is a flowering activator in rice (Lee *et al*., 2004), it is evident that a functional differentiation event occurred between the orthologous genes *TaSOC1* and *OsMADS50*. Surprisingly, no *TaSOC1* orthologue was detected in barley, a relative of wheat (Figure S4c). We also identified two other groups of *SOC1‐like* genes, designated as *TaSOC1‐like1* and *TaSOC1‐like2*, on wheat homoeologous group‐1 chromosomes. Genome assembly showed that *TaSOC1‐like2* (*TraesCS1A02G199900* and *TraesCS1D02G203400*) were derived from *TaSOC1‐like1* (*TraesCS1A02G199600*, *TraesCS1B02G214500* and *TraesCS1D02G203300*) through tandem duplication events. Synteny analysis revealed that the *TaSOC1‐like* genes had genomic collinearity with *OsMADS56* (Figure S4d). The responses of *TaSOC1‐like* genes to photoperiod and vernalization treatments were also investigated. Like *TaSOC1*, the *TaSOC1‐like1* and *TaSOC1‐like2* genes were repressed by vernalization although the latter exhibited weaker responses than the former. However, SD could not induce the expression of the *TaSOC1‐like* genes. Additionally, *TaSOC1‐like* genes were repressed by SD, which is reverse of the response of *TaSOC1* to photoperiod treatment (Figure S5). *HvSOC1‐like1*, a *SOC1* homologue in barley and orthologue to *OsMADS56* in rice, was induced by vernalization (Figure S4d) (Papaefthimiou *et al*., 2012). *HvSOC1‐like1* was also orthologous to *TaSOC1‐like* genes in wheat (Figure S4d). Overall, *SOC1* homologues in wheat have expanded greatly and undergone considerable functional differentiation although all of them are probably involved in flowering regulation. It is worth exploring the specific functions of each *TaSOC1‐like* gene to dissect the flowering regulatory mechanisms in wheat.

### The molecular network of *TaSOC1*‐modulated flowering through integration of the vernalization and photoperiod pathways

Understanding the plasticity of genetic architecture underlying flowering time is prerequisite to design resilient crops. *SOC1* is an integrator of several flowering pathways, including photoperiod, temperature, hormone and plant age (Lee and Lee, 2010). Here, we confirmed that the wheat *SOC1* homologue *TaSOC1* also integrated photoperiod and vernalization signals to modulate flowering, suggesting that this gene plays an essential role in response to environmental cues to initiate flowering. TaSOC1 directly interacted with TaVRN1 to repress flowering in the vernalization pathway. A previous study showed that TaVRN1 could achieve positive feedback self‐regulation through binding with TaVRT2 (Xie *et al*., 2021). Yeast three‐hybrid (Y3H) assays revealed that TaSOC1 could disrupt interaction between TaVRT2 and TaVRN1, and TaVRT2 could also disrupt the TaVRN1‐TaSOC1 interaction (Figure S6). This result indicates that TaSOC1 and TaVRT2 competitively bind to TaVRN1. *Flowering promoting factor 1* (*FPF1*) genes were reported to accelerate flowering in *Arabidopsis*. Transcriptome assays showed that the *FPF1*‐like gene *TaFPF1‐2B* was repressed by *TaSOC1* and induced by *TaVRN1* (Figure 5b–d; Dataset S6). Moreover, TaSOC1 and TaVRN1 were enriched at certain sites close to the TSS motifs in *TaFPF1‐2B* (Figure 5f). Thus, *TaFPF1‐2B* could function as a common downstream gene of *TaSOC1* and *TaVRN1* to modulate flowering. To further identify the relationship among *TaSOC1*, *TaVRN1*, *TaVRT2* and *TaFPF1* in flowering initiation, we investigated changes in expression of these genes in response to pre‐flowering vernalization and photoperiod patterns simulating temperature and photoperiod conditions in the field (Figure S7a). Based on results, we proposed a regulatory model of *TaSOC1* in orchestrating *TaVRN1*, *TaVRT2* and *TaFPF1* to modulate flowering through integration of photoperiod and vernalization signals (Figure S7b,c). According to the model, *TaVRN1* in winter wheat is expressed at very low levels after germination in the fall and cannot promote expression of *TaFPF1*. *TaVRT2* is gradually upregulated during cold winter temperatures (vernalization) and subsequently enhances expression of *TaVRN1* (Xie *et al*., 2021). The increased transcript abundance of *TaVRN1* induces expression of *TaFPF1*. However, *TaSOC1* is up‐regulated by short photoperiod in winter and thus represses expression of *TaFPF1*. Accordingly, *TaFPF1* still exhibits low expression level in winter due to the combined effects of *TaSOC1*, *TaVRN1* and *TaVRT2*. With increasing daylength and temperature in spring, *TaVRT2* expression sharply declines, leading to a reduction in *TaVRN1* expression. Soon afterwards, *TaVRN1* expression continues to increase due to its self‐regulatory loop mediated by the TaVRN3/TaFDL2 complex (Chen and Dubcovsky, 2012; Deng *et al*., 2015; Li and Dubcovsky, 2008). Concurrently, *TaSOC1* expression is dramatically down‐regulated and then has a modest increase. However, *TaSOC1* remains a much lower expression level than *TaVRN1*, causing *TaFPF1* to be rapidly up‐regulated and ultimately allowing concomitant flower initiation. Overall, TaSOC1, TaVRN1, TaVRT2 and TaFPF1 form a dynamic interactive network to control wheat flowering in response to photoperiod and vernalization. Among them, *TaSOC1* acts as an integrator to coordinate cross‐talk between the photoperiod and vernalization flowering pathways.

This study confirmed that *TaSOC1* expression was regulated by both photoperiod and vernalization, although the underlying regulatory mechanism remained largely unknown. *SOC1* can be activated by *CONSTANS* (*CO*) through FT in long photoperiods and repressed by *FLC*, a core component of the vernalization pathway in *Arabidopsis* (Lee and Lee, 2010). The wheat homologues of *FT*, *CO* and *FLC* have conserved function in flowering time, so their roles in regulating *TaSOC1* expression should be investigated (Li *et al*., 2011; Nemoto *et al*., 2003; Sharma *et al*., 2017; Shaw *et al*., 2020; Yan *et al*., 2006). *Ppd‐1* is a key gene in the wheat photoperiod flowering pathway (Shaw *et al*., 2012, 2013). *Phytochrome C* is another major regulator to promote flowering under LD conditions in wheat (Chen *et al*., 2014). *TaVRN2* is involved in the vernalization through *TaVRN1* and its expression is repressed by SD (Dubcovsky *et al*., 2006; Li *et al*., 2011; Trevaskis *et al*., 2006; Yan *et al*., 2004b). Thus, it is necessary to identify the relationship of *TaSOC1* with *Ppd‐1*, *Phytochrome C* and *TaVRN2* in the photoperiod pathway. Overall, *TaSOC1* is an important checkpoint in dissecting regulatory network underlying wheat flowering in response to environmental cues.

## Materials and methods

### Plant materials, growth conditions and flowering time measurement

KN199, an elite semi‐winter wheat cultivar, needs approximately 4 weeks at low temperature (2–6 °C) to fully meet its vernalization requirement. KN199 was used as a transgenic recipient and grown with the transgenic lines in a greenhouse under normal conditions (15–18 °C, 16 h light/8 h dark). For vernalization treatments, 3‐week‐old seedlings were moved into a cold room at 2–6 °C for 30 days (complete vernalization) or 14 days (incomplete vernalization). Photoperiodic treatments of long daylight (16 h light/8 h dark) and short daylight (8 h light/16 h dark) were applied to fully vernalized three‐leaf seedlings for 3 weeks. For gibberellin treatments, 3‐week‐old plants were sprayed with a solution containing 0.1 mM GA_3_ (Yuan Ye Biotech, Shanghai, China) and 0.05% Tween 20 (BIORIGIN, Beijing) once a week for 5 weeks; water with 0.05% Tween 20 acted as the control. A diverse panel of 166 elite wheat cultivars from the Yellow and Huai River Valleys, the largest wheat‐producing region in China, were used to validate the genetic effects of the genes of interest (Datasets S10 and S11). All lines were planted in six environments (Dataset S11). The field trial in each environment was conducted in a completely randomized block design with three replications and a two‐row plot with 2 m row length and 20 cm row spacing.

Wheat flowering time was evaluated as heading date which was measured as the number of days from seed germination to heading of the main stem. Approximately, 40 plants of each line were used for statistical analyses. Heading date data for the diversity panel were available from Li *et al*. (2019).

### LCI and BiFC experiments

*Nicotiana benthamiana* was grown in a growth chamber (20–23 °C, 16 h light/8 h dark) for LCI experiments. LCI assays were carried out as described previously (He *et al*., 2019). The coding sequences (CDSs) of *TaVRN1* (GenBank accession number JQ915056) and *TaSOC1* (GenBank accession number AM502888) were cloned into vectors pCAMBIA1300‐nLUC and pCAMBIA1300‐cLUC, respectively, which were then co‐transformed into *N. benthamiana* leaves by agroinfiltration. LUC activities in the leaves were measured at 50 h post infiltration. The infiltrated leaves were sprayed with 100 μL luciferase assay substrate (Promega, Beijing) and imaged using LB985 NightSHADE (Berthhold Technologies, Bad Wildbad, Germany). Sample analyses were based on at least three biological replicates.

For BiFC analysis, the CDSs of *TaVRN1* and *TaSOC1* were cloned into pUC‐SPYNE(R)173 and pUC‐SPYCE(M), respectively. Wheat protoplasts from young leaves of KN199 were prepared and transfected as described in Shan *et al*. (2014). Fluorescence signals of yellow fluorescent protein (YFP) were analysed with a confocal laser scanning microscope (LSM710, Carl Zeiss). Primers are summarized in Dataset S12.

### RT‐qPCR analyses

Total RNA was extracted using an EasyPure Plant RNA Kit (ER301; Transgene, Beijing), and first‐strand cDNA was synthesized using a PrimeScript RT Reagent Kit with gDNA Eraser (RR047A; TaKaRa, Dalian) according to the manufacturer's instructions. The RT‐qPCR mixture comprised 3 μL cDNA template, 0.75 μL of each primer (10 μM) and 7.5 μL 2× Universal SYBR Green Fast qPCR Mix (RK21203; ABclonal, Wuhan) in a final volume of 15 μL. Amplification was performed on a BioRad CFX system following the manufacturer's protocol. The expression levels of target genes were normalized to a wheat *actin* gene (GenBank accession number AB181991) and calculated by the 2^−ΔΔCt^ method (Schmittgen and Livak, 2008). Each sample was analysed as three biological replicates. The primers used for RT‐qPCR are listed in Dataset S12.

### Transgenic experiments

For the generation of *TaSOC1* overexpression transgenic plants, the CDS of *TaSOC1* was cloned into the entry vector pDONR207 and then transferred into the destination vector pUbiGW following the handbook for Gateway cloning (Invitrogen, Carlsbad, CA). The resultant construct was transformed into immature embryos of KN199 by the *Agrobacterium* (strain EHA105)‐mediated method (Ishida *et al*., 2015). *TaVRN1* overexpression lines in this study were generated by Xie *et al*. (2021).

The CRISPR/SpCas9 system was used to produce knockout mutant of *TaSOC1*. The small guide RNAs sgRNA1 (PAM‐guide sequence 5′‐CCTTCTAGGCATCAGGGCAATGA‐3′) and sgRNA2 (PAM‐guide sequence 5′‐CCTTCTAGGCATCAGGGCAGTGA‐3′) were designed to target the conserved regions in the fourth exons of *TaSOC1* and its orthologues *TaSOC1‐5A* and *TaSOC1‐4D*. Both sgRNAs were cloned into the vector pWMBX110‐SpCas9 (Liu *et al*., 2020). The resulting constructs were introduced into KN199 via *Agrobacterium* (strain EHA105)‐mediated transformation (Ishida *et al*., 2015).

### RNA‐seq assays

Samples used for RNA‐seq were prepared from leaves of transgenic lines and their TNL at the stem elongation stage (from double ridge to floret differentiation), with three biological replicates. RNA extraction, library construction and sequencing were carried out by Novogene (Beijing). A total 10 Gb of transcriptomic data for each sample was obtained from the Illumina HiSeq 4000 platform. Differentially expressed genes (DEGs) were defined according to |fold change| >2 and *P* < 0.01.

### ChIP‐qPCR systems

ChIP‐qPCR assays were performed according to the protocol from Cao *et al*. (2014) with a little modification. The young leaves from 3‐week‐old seedling of KN199 were harvested. The leaf tissue (1.5 g) was ground in liquid nitrogen and fully resuspended in 30 mL nuclei isolation buffer (10 mM HEPES pH 7.6, 400 mM sucrose, 5 mM KCl, 5 mM MgCl_2_, 5 mM EDTA, 1% formaldehyde, 14.4 mM 2‐mercaptoethanol, 0.6% Triton X‐100 and 0.4 mM PMSF). The crosslinking reaction in the nuclei was carried out at room temperature for 10 min and stopped by 2 mL 2 M glycine. The nuclei were filtered, centrifuged at 2800 ***g*** for 15 min and resuspended in 220 μL nuclei lysis buffer (50 mM Tris–HCl pH 8.0, 1.0% SDS, 10 mM EDTA pH 8.0). The chromatin released from nuclei was sonicated by the Bioruptor Pico device (Diagenode) at 15 s on/90 s off for seven cycles and centrifuged at 20 000 ***g*** for 5 min. The supernatant containing sheared chromatin was equally dispensed into two fresh tubes and each was adjusted to 1.1 mL with ChIP dilution buffer (0.01% SDS, 1.1% TritonX‐100, 1.2 mM EDTA, 16.7 mM Tris–HCl PH 8.0, 167 mM NaCl). Resultant solution (100 μL) from each tube was transferred into a clean tube and stored at −20 °C as an input. Subsequently, 40 μL protein A + G magnetic beads (Cat#16‐663; Merck Millipore) together with 2 μg GFP antibody (Cat#ab290; Abcam) or without antibody as negative control (NC) were added to the tubes containing sheared chromatin; the mixtures were shaken overnight at 4 °C. After incubation, the beads were repeatedly washed in 1 mL low‐salt immune complex wash buffer (0.1% SDS, 1.0% Triton X‐100, 2 mM EDTA, 20 mM Tris–HCl pH 8.0, 150 mM NaCl), 1 mL high‐salt immune complex buffer (0.1% SDS, 1.0% Triton X‐100, 2 mM EDTA, 20 mM Tris–HCl pH 8.0, 500 mM NaCl), 1 mL LiCl immune complex wash buffer (0.25 M LiCl, 1.0% NP‐40, 1.0% sodium dexycholate, 1 mM EDTA, 10 mM Tris–HCl pH 8.0) and 1 mL TE, and eluted by 200 μL elution buffer (1.0% SDS, 100 mM NaHCO_3_). The eluate was mixed with 20 μL of 5 M NaCl and de‐crosslinked by incubation overnight at 65 °C. Afterwards, 4 μL of 0.5 M EDTA, 8 μL of 1 M Tris–HCl (pH 6.5) and 2 μL of proteinase K (20 mg/mL; Fermentas, Shanghai) were incubated with the de‐crosslinked eluate for 2 h at 45 °C. The DNA was extracted from the eluate with chloroform/isoamyl alcohol (24:1) and precipitated with an equal volume of anhydrous ethanol in the presence of 0.3 M sodium acetate (pH 5.2) and 5 μL glycogen (20 mg/mL; Macklin, Shanghai, China). The DNA pellet was washed with 70% ethanol and then dissolved in 50 μL TE. The enrichment of co‐immunoprecipitated DNA was calculated by the 2^−ΔΔCt^ method (Schmittgen and Livak, 2008). Primers are listed in Dataset S12.

### Y1H and Y3H assays

Y1H was used to validate the interaction of TaSOC1 and TaVRN1 with representative sites 5, 7, 8 and 9 close to the *TaFPF1‐2B* transcription start site. Each CDS of *TaSOC1* and *TaVRN1* was inserted into the pB42AD vector (Cat#ZT0295, Clontech), respectively. Each of the sites was cloned into the pLacZi reporter vector (Cat#631707, Clontech). The resultant pB42AD and pLacZi vectors were co‐transformed into the yeast strain EGY48 using the lithium acetate method following the user manual in Clontech (https://www.takarabio.com/documents/User%20Manual/PT3024/PT3024‐1.pdf). Transformants were selected on SD‐Trp/Ura plates at 30 °C for 2–4 days and positive transformants were transferred to X‐Gal (5‐bromo‐4‐chloro‐3‐indolyl‐b‐d‐galactopyranoside) plates for galactosidase activity analysis. Primers for Y1H are listed in Dataset S12.

Y3H assays were used to test whether TaSOC1 can interfere with the interaction between TaVRT2 and TaVRN1. The CDSs of *TaVRT2* (GenBank accession number AAY43789) and *TaSOC1* were inserted in MCS I or MCS II, respectively, of the pBridge vector (TaKaRa, Dalian), while that of *TaVRN1* was fused with the pGADT7 vector (TaKaRa). The resulting constructs and the empty vectors (used as controls) were co‐transferred into yeast AH109 cells using the lithium acetate method. Transformants were selected on the synthetic defined (SD) medium without leucine (L) and tryptophan (W) (SD‐L/W). Positive transformants were used to identify the interaction among the proteins of interest on the SD mediums without L, W and histidine (H) (SD‐L/W/H) or without L, W, H and methionine (M) (SD‐L/W/M/H). The detailed procedure of yeast transformation and protein interaction assays were performed as described in the Yeast Protocols Handbook (https://www.takarabio.com/documents/User%20Manual/PT3024/PT3024‐1.pdf). Primers for Y3H are listed in Dataset S12.

### Identification of allelic variations and development of gene‐specific markers

Sequence variants of the genes of interest were identified from the WheatUnion databases (Guo *et al*., 2020; http://wheat.cau.edu.cn/WheatUnion/). Haplotypes of target genes were defined by the genomic variation information of 100 modern Chinese cultivars (Hao *et al*., 2020; Dataset S9). Gene‐specific markers were developed to genotype allelic variations. Genotypic and phenotypic data of the 166 elite wheat cultivars from the Yellow and Huai River Valleys of China were used for association analysis to mine superior haplotypes.

### Statistical analyses

Student's *t*‐tests were performed to assess significant differences (*P* < 0.05) in flowering time, gene expression levels and chromatin enrichment between contrasting materials using SAS 9.4. Epistatic effects of *TaSOC1* and *TaVRN1* on flowering time were analysed by two‐way ANOVA in SAS 9.4. Mean values and error bars were calculated with the AVERAGE and STDEV functions, respectively, in Microsoft Excel.

## Conflict of interest

The authors declare no conflicts of interest.

## Author contributions

X.M.L., B.Y.L., L.X., K.W., D.A.X., X.L.T., L.N.X. and L.L.L. performed the experiments; S.H.C. designed the experiment; S.H.C. and X.M.L. wrote the draft; L.L.Y., X.C.X., Z.H.H. and X.G.Y. revised the manuscript.

## Supporting information


**Figure S1** Expression patterns of *TaSOC1* (*TraesCS4B02G346700*) (a) and *TaVRN1* (*TraesCS5A02G391700*) (b) in different tissues according to Wheat Expression Browser (http://wheat‐expression.com/). The Wheat Expression Browser (http://wheat‐expression.com/) is a powerful platform for analyses of gene expression patterns across tissues and developmental time courses, and includes more than 850 RNA‐seq datasets from many wheat cultivars or lines, such as Chinese Spring, Azhurnaya, Riband, Vuka, Avocet, specific near‐isogenic lines and some synthetic hexaploid wheat.
**Figure S2** ChIP‐qPCR detection sites in *TaFPF1* promoter. CArG‐like motifs within the promoter of 2 kb are shown in red and the target fragments of qPCR are underlined.
**Figure S3** *TaVRN1* response to photoperiod treatments. (a) The expression pattern of *TaVRN1* under different photoperiod treatments (*n* = three biological replicates). The numbers on the horizontal axis represent sampling time‐points: 1, day before photoperiod treatments; 2–5, the 5th, 10th, 15th and 20th day during photoperiod treatments; 6–7, the 5th and 10th day after photoperiod treatments. Orange and blue lines represent short‐day (SD) and long‐day (LD) treatments, respectively. (b) Expression pattern of *TaVRN1* at different time‐points in a single day under SD and LD treatments (*n* = three biological replicates). 1–5 represent time‐points 6:00, 10:00, 14:00, 18:00 and 22:00, respectively; SD and LD responses are shown in orange and blue, respectively. Statistical analyses (c) and phenotypes (d) of heading date for *TaVRN1* overexpression lines (*TaVRN1*‐OE) and transgenic null lines (TNL) under photoperiod treatments. ns, not significant; scale bar, 30 cm.
**Figure S4** Phylogenetic and synteny analyses of wheat *SOC1* homologues. (a) Phylogenetic analysis of *TaSOC1* and its homologues across major cereal crops and *Arabidopsis thaliana* (AT). BRADI, *Branchpodium distachyon*; HORVU, *Hordeum vulgare* (barley); Os, *Oryza sativa* (rice); Traes, *Triticum aestivum* (bread wheat); Zm, *Zea mays* (maize); T, tandem duplication. (b) Synteny analyses of *TaSOC1* and its orthologues *TaSOC1‐5A* and *TaSOC1‐4D* with rice *OsSOC1*/*OsMADS50*. (c) Gene collinearity of the wheat genomic region containing *TaSOC1* with the counterparts in three barley cultivars. The genomes of wheat and barley cultivars are labelled in the left panel. Red arrowhead shows *TaSOC1*. (d) Synteny comparison of *TaSOC1‐like* genes with rice *OsMADS56* and barley *HvSOC1‐like1*.
**Figure S5** Responses of *TaSOC1‐like* genes to vernalization (a) and photoperiod (b). In (a): V0, the day before vernalization; V7, V21 and V35, 7th, 21st and 35th day during vernalization; V35N7 and V35N21 indicate the 7th and 21st day after vernalization, respectively; orange and blue lines represent vernalized and non‐vernalized (negative control) treatments, respectively. In (b), sampling time‐points include the day before photoperiod treatments (1), 5th (2), 10th (3), 15th (4) and 20th (5) day during photoperiod treatments, and 5th (6) and 10th (7) day after photoperiod treatment; orange and blue lines represent short‐day (SD) and long‐day (LD) treatments, respectively; error bars, standard deviations of three biological replicates.
**Figure S6** Yeast three hybrid arrays for competitive interaction of TaSOC1 and TaVRT2 with TaVRN1. SD‐L/W, SD‐L/W/H, SD‐L/W/M/H represent the plates with synthetic defined (SD) media lacking Leu/Trp, Leu/Trp/His, Leu/Trp/Met/His, respectively; 10^0^, 10^−1^, 10^−2^ and 10^−3^ indicate gradient dilution of yeast concentration. AD, activation domain; BD, DNA binding domain.
**Figure S7** Molecular model of wheat flowering synergistically modulated by *TaSOC1*, *TaVRN1*, *TaVRT2* and *TaFPF1*. (a) Expression analyses of *TaSOC1*, *TaVRN1*, *TaVRT2* and *TaFPF1* under simulated winter conditions using quantitative real‐time PCR (*n* = three biological replicates). The orange and blue lines represent short‐day (SD) (simulated winter) and long‐day (LD) (control group) treatments during winter (low temperature), respectively; LD were applied before and after winter. The numbers on the abscissa represent the sampling time points, including the 10th (BV1) and 5th (BV2) day before winter, the 5th (VSD5), 10th (VSD10), 15th (VSD15) and 20th (VSD20) day during winter, and the 5th (NS5), 10th (NS10), 15th (NS15) and 20th (NS20) day after winter. (b) Schematics of the dynamic expression abundances of *TaSOC1*, *TaVRN1*, *TaVRT2* and *TaFPF1* before, during and after winter. (c) Molecular model of *TaSOC1*, *TaVRN1* and *TaVRT2* modulating *TaFPF1* expression. Colour intensity indicates the level of protein accumulation and arrow thickness represents the transcriptional activity of *TaFPF1*. Note: single TaVRN1 or TaSOC1 as well as their homo‐dimers are possible to bind to the *TaFPF1* promoter.

**Dataset S1** Statistical analysis on the heading date of TaSOC1‐OE and TNL under different vernalization conditions.
**Dataset S2** Statistical analysis on the heading date of TaSOC1‐KO and NC under different vernalization conditions.
**Dataset S3** Statistical analysis of the heading date of segregating population from the cross of TaSOC1‐OE and TaVRN1‐OE lines.
**Dataset S4** Differentially expressed genes between TaSOC1 overexpression lines and their transgenic negative lines.
**Dataset S5** Differentially expressed genes between TaVRN1 overexpression lines and their transgenic negative lines.
**Dataset S6** Overlapping downstream differentially expressed genes of TaSOC1 and TaVRN1.
**Dataset S7** Statistical analysis on the heading date of TaSOC1‐OE and TNL under GA or photoperiod treatments.
**Dataset S8** Statistical analysis on the heading date of TaSOC1‐KO and NC under different photoperiod treatments.
**Dataset S9** Identification for the haplotypes of TaSOC1 and TaSOC1‐5A based on resequencing data of 100 Chinese wheat cultivars.
**Dataset S10** Haplotypes of TaSOC1 and TaSOC1‐5A, and heading date of 166 wheat cultivars from the Yellow and Huai River Valleys.
**Dataset S11** Association analyses of TaSOC1 and TaSOC1‐5A haplotypes with heading date.
**Dataset S12** The list of primers used in this study.
