# Supplementary material for: The TaSOC1‐TaVRN1 module integrates photoperiod and vernalization signals to regulate wheat flowering
Source: Plant Biotechnol J. 2023 Nov 8;22(3):635–49. doi: 10.1111/pbi.14211 (PMC10893938; doi:10.1111/pbi.14211)
Supplement: Supplementary file 1 — Figure S1 Expression patterns of TaSOC1 (TraesCS4B02G346700) (a) and TaVRN1 (TraesCS5A02G391700) (b) in different tissues according to Wheat Expression Browser (http://wheat‐expression.com/). The Wheat Expression Browser (http://wheat‐expression.com/) is a powerful platform for analyses of gene expression patterns across tissues and developmental time courses, and includes more than 850 RNA‐seq datasets from many wheat cultivars or lines, such as Chinese Spring, Azhurnaya, Riband, Vuka, Avocet, specific near‐isogenic lines and some synthetic hexaploid wheat. Figure S2 ChIP‐qPCR detection sites in TaFPF1 promoter. CArG‐like motifs within the promoter of 2 kb are shown in red and the target fragments of qPCR are underlined. Figure S3 TaVRN1 response to photoperiod treatments. (a) The expression pattern of TaVRN1 under different photoperiod treatments (n = three biological replicates). The numbers on the horizontal axis represent sampling time‐points: 1, day before photoperiod treatments; 2–5, the 5th, 10th, 15th and 20th day during photoperiod treatments; 6–7, the 5th and 10th day after photoperiod treatments. Orange and blue lines represent short‐day (SD) and long‐day (LD) treatments, respectively. (b) Expression pattern of TaVRN1 at different time‐points in a single day under SD and LD treatments (n = three biological replicates). 1–5 represent time‐points 6:00, 10:00, 14:00, 18:00 and 22:00, respectively; SD and LD responses are shown in orange and blue, respectively. Statistical analyses (c) and phenotypes (d) of heading date for TaVRN1 overexpression lines (TaVRN1‐OE) and transgenic null lines (TNL) under photoperiod treatments. ns, not significant; scale bar, 30 cm. Figure S4 Phylogenetic and synteny analyses of wheat SOC1 homologues. (a) Phylogenetic analysis of TaSOC1 and its homologues across major cereal crops and Arabidopsis thaliana (AT). BRADI, Branchpodium distachyon; HORVU, Hordeum vulgare (barley); Os, Oryza sativa (rice); Traes, Triticum aestivum (bre [file PBI-22-635-s002.docx]

**Supplemental figures**

**
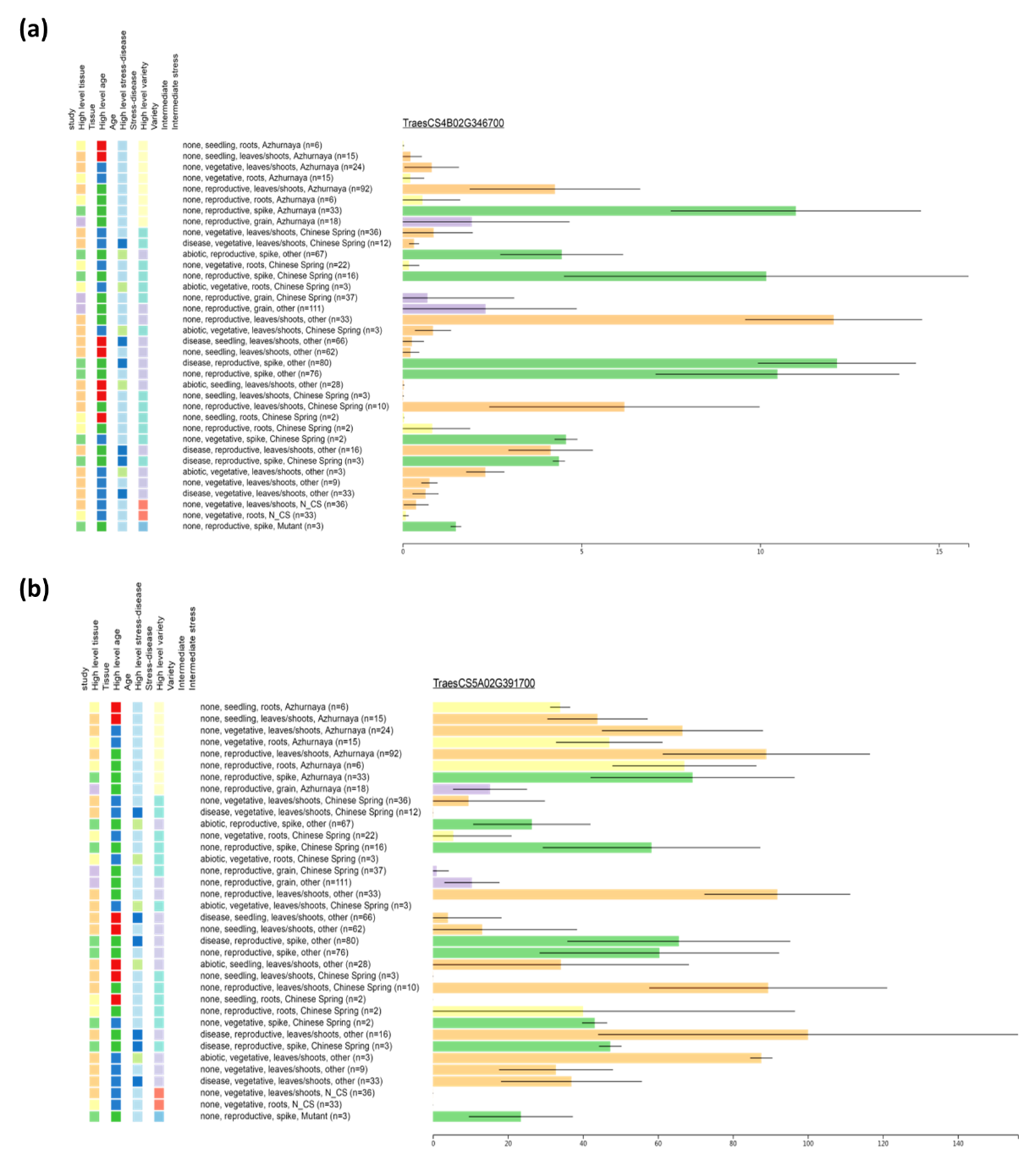
**

**Figure S1 Expression patterns of *TaSOC1* (*TraesCS4B02G346700*) (a) and *TaVRN1* (*TraesCS5A02G391700*) (b) in different tissues according to Wheat Expression Browser (**[**http://wheat-expression.com/**](http://wheat-expression.com/)**)**

The Wheat Expression Browser (http://wheat-expression.com/) is a powerful platform for analyses of gene expression patterns across tissues and developmental time courses, and includes more than 850 RNA-seq datasets from many wheat cultivars or lines, such as Chinese Spring, Azhurnaya, Riband, Vuka, Avocet, specific near-isogenic lines and some synthetic hexaploid wheat.

**
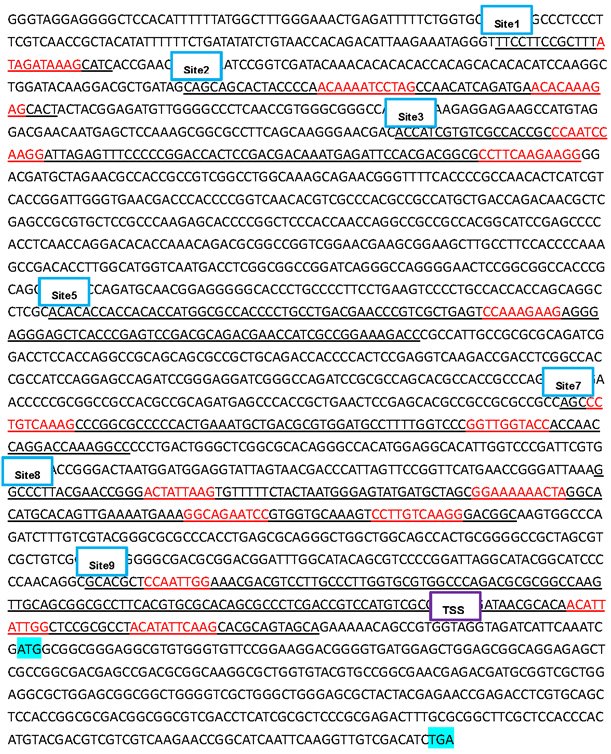
Figure S2 ChIP-qPCR detection sites in *TaFPF1* promoter**

CArG-like motifs within the promoter of 2 kb are shown in red and the target fragments of qPCR are underlined.

**
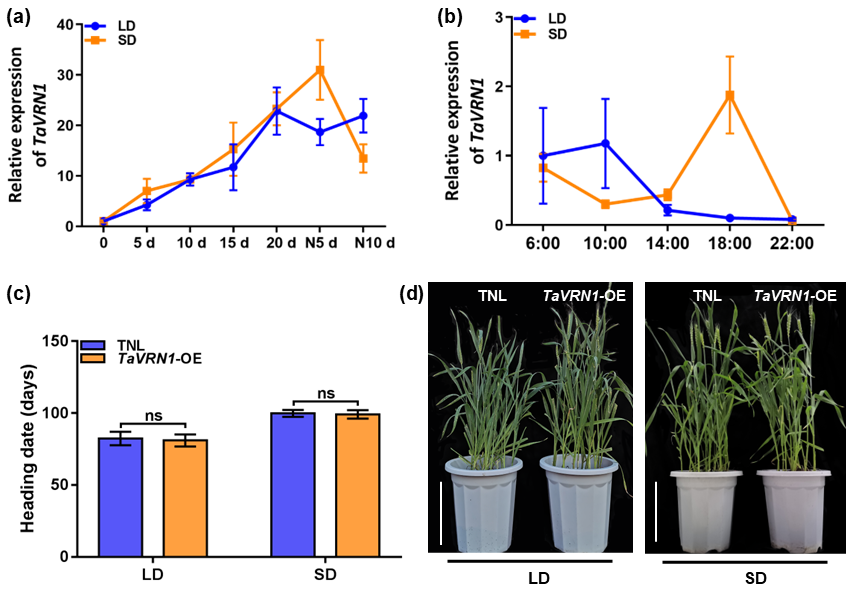
Figure S3 *TaVRN1* response to photoperiod treatments**

**(a)** The expression pattern of *TaVRN1* under different photoperiod treatments (n = three biological replicates). The numbers on the horizontal axis represent sampling time-points: 1, day before photoperiod treatments; 2-5, the 5^th^, 10^th^, 15^th^ and 20^th^ day during photoperiod treatments; 6-7, the 5^th^ and 10^th^ day after photoperiod treatments. Orange and blue lines represent short-day (SD) and long-day (LD) treatments, respectively. **(b)** Expression pattern of *TaVRN1* at different time-points in a single day under SD and LD treatments (n = three biological replicates). 1-5 represent time-points 6:00, 10:00, 14:00, 18:00 and 22:00, respectively; SD and LD responses are shown in orange and blue, respectively. Statistical analyses **(c)** and phenotypes **(d)** of heading date for *TaVRN1* overexpression lines (*TaVRN1*-OE) and transgenic null lines (TNL) under photoperiod treatments. ns, not significant; scale bar, 30 cm.

**
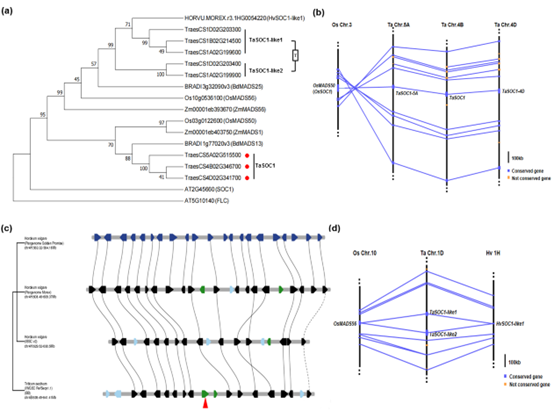
Figure S4 Phylogenetic and synteny analyses of wheat *SOC1* homologs**

**(a)** Phylogenic analysis of *TaSOC1* and its homologs across major cereal crops and *Arabidopsis* *thaliana* (AT). BRADI, *Branchpodium distachyon*; HORVU, *Hordeum vulgare* (barley); Os, *Oryza sativa* (rice); Traes, *Triticum aestivum* (bread wheat); Zm, *Zea mays* (maize); T, tandem duplication. **(b)** Synteny analyses of *TaSOC1* and its orthologs *TaSOC1-5A* and *TaSOC1-4D* with rice *OsSOC1*/*OsMADS50*. **(c)** Gene collinearity of the wheat genomic region containing *TaSOC1* with the counterparts in three barley cultivars. The genomes of wheat and barley cultivars are labeled in the left panel. Red arrowhead shows *TaSOC1*. **(d)** Synteny comparison of *TaSOC1-like* genes with rice *OsMADS56* and barley *HvSOC1-like1*.

**
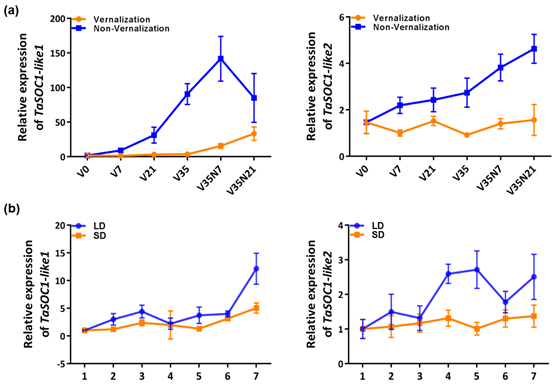
Figure S5 Responses of *TaSOC1-like* genes to vernalization (a) and photoperiod (b)**

In **(a)**: V0, the day before vernalization; V7, V21 and V35, 7^th^, 21^st^ and 35^th^ day during vernalization; V35N7 and V35N21 indicate the 7^th^ and 21^st^ day after vernalization, respectively; orange and blue lines represent vernalized and non-vernalized (negative control) treatments, respectively. In **(b)**, sampling time-points include the day before photoperiod treatments (1), 5^th^ (2), 10^th^ (3), 15^th^ (4) and 20^th^ (5) day during photoperiod treatments, and 5^th^ (6) and 10^th^ (7) day after photoperiod treatment; orange and blue lines represent short-day (SD) and long-day (LD) treatments, respectively; error bars, standard deviations of three biological replicates.

**
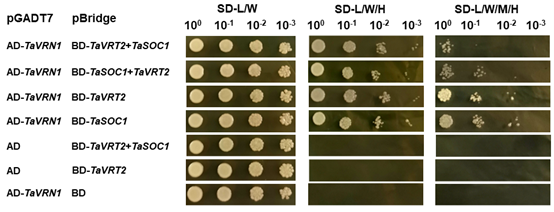
****Figure S6 Yeast three hybrid arrays for competitive interaction of TaSOC1 and TaVRT2 with TaVRN1**

SD-L/W, SD-L/W/H, SD-L/W/M/H represent the plates with synthetic defined (SD) media lacking Leu/Trp, Leu/Trp/His, Leu/Trp/Met/His, respectively; 10^0^, 10^-1^, 10^-2^ and 10^-3^ indicate gradient dilution of yeast concentration. AD, activation domain; BD, DNA binding domain

**
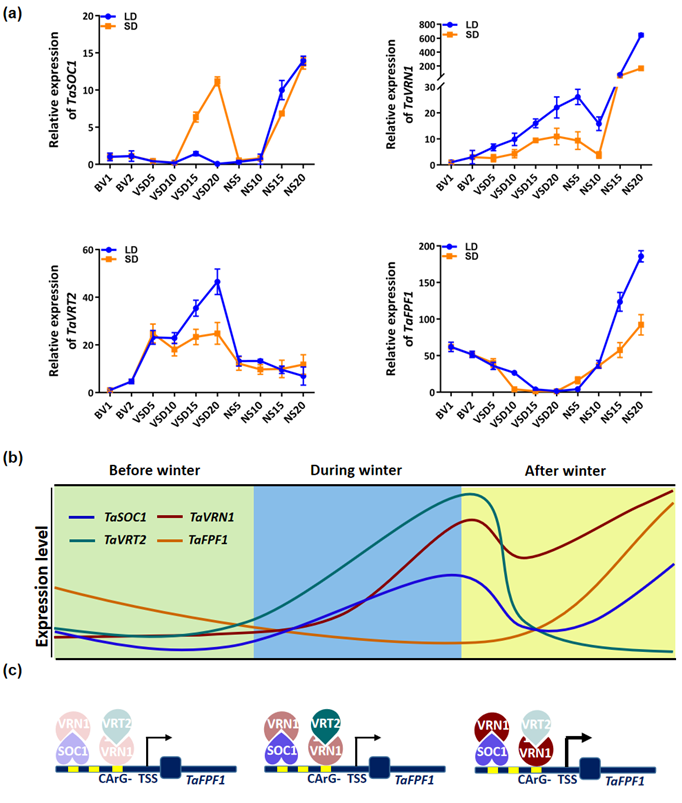
Figure S7 Molecular model of wheat flowering synergistically modulated by *TaSOC1*, *TaVRN1*, *TaVRT2* and *TaFPF1***

**(a)** Expression analyses of *TaSOC1*, *TaVRN1*, *TaVRT2* and *TaFPF1* under simulated winter conditions using quantitative real-time PCR (n = three biological replicates). The orange and blue lines represent short-day (SD) (simulated winter) and long-day (LD) (control group) treatments during winter (low temperature), respectively; LD were applied before and after winter. The numbers on the abscissa represent the sampling time points, including the 10^th^ (BV1) and 5^th^ (BV2) day before winter, the 5^th^ (VSD5), 10^th^ (VSD10), 15^th^ (VSD15) and 20^th^ (VSD20) day during winter, and the 5^th^ (NS5), 10^th^ (NS10), 15^th^ (NS15) and 20^th^ (NS20) day after winter. **(b)** Schematics of the dynamic expression abundances of *TaSOC1*, *TaVRN1*, *TaVRT2* and *TaFPF1* before, during and after winter. **(c)** Molecular model of *TaSOC1*, *TaVRN1* and *TaVRT2* modulating *TaFPF1* expression. Color intensity indicates the level of protein accumulation and arrow thickness represents the transcriptional activity of *TaFPF1.*
